# Spontaneous unscarred uterine rupture at 13 weeks of gestation after in vitro fertilization-embryo transfer: A case report and literature review

**DOI:** 10.1097/MD.0000000000036254

**Published:** 2023-12-08

**Authors:** Juan Zheng, Liming Zhou, Juwei Hu

**Affiliations:** a Reproductive Medicine Center, Ningbo Women and Children’s Hospital, Ningbo City, Zhejiang Province, China.

**Keywords:** hysterectomy, in vitro fertilization embryo transfer, systemic lupus erythematosus, uterine rupture

## Abstract

**Rationale:**

Uterine rupture (UR) during pregnancy is a serious obstetric complication. Here we report a case of spontaneous rupture in an unscarred uterus at 13 weeks of gestation after in vitro fertilization embryo transfer, which is not common in past references. Our focus is to understand the relationship between systemic lupus erythematosus (SLE) and UR.

**Patient concerns:**

A 33-year-old infertile woman with a history of SLE became pregnant after in vitro fertilization embryo transfer. She presented with sudden mental fatigue and dyspnea, accompanied by sweating, dizziness and lower abdominal pain at 13 weeks of gestation.

**Diagnoses:**

Blood analysis revealed anemia. Ultrasonography and plain computed tomography scan revealed intrauterine early pregnancy with effusion in pelvic and abdominal cavity. Laparotomy confirmed the diagnosis of UR.

**Interventions:**

The patient underwent emergency laparotomy. Upon surgery, multiple myometrium was weak with only serosal layer visible, and there was a 2.5 cm irregular breach with exposed placenta and villous tissue in the posterior wall of the uterus. After removing intrauterine fetus and repairing the breach, there was still persistent intraperitoneal hemorrhage. The patient underwent subtotal hysterectomy finally.

**Outcomes:**

Postoperative recovery was uneventful. The patient was discharged on the 8th day after operation.

**Lessons:**

Combined efforts of specialists from ultrasound, imaging and gynecologist led to the successful diagnosis and management of this patient. We should be cautious about the occurrence of unscarred uterus rupture during pregnancy of the women with the disease of SLE and long-term glucocorticoid treatment. In IVF, we had better transfer one embryo for these patients with the history of SLE. Obstetricians should strengthen labor tests to detect early signs of UR of the patients with SLE and long term glucocorticoid treatment. Once UR is suspected, prompt surgical treatment is needed as soon as possible.

## 1. Introduction

Uterine rupture (UR) during pregnancy is a life-threatening complication. Scarred UR is not difficult to diagnose. However, it is not easy to identify unscarred uterine rupture (UUR) because the risk factors are complex. According to literature review, the risk factors include high parity (≥4 births), uterine anomalies or diverticula, fetal macrosomia, multiple gestations, abnormal placentation, uterine adenomyosis, curettage, underlying connective tissue disorder and injudicious use of oxytocin.^[1–11]^ Here, we present a rare case of UUR in a primiparous woman at 13 weeks of gestation after in vitro fertilization embryo transfer who was diagnosed with the disease of systemic lupus erythematosus (SLE).

## 2. Case report

A 33-year-old gravida occurred sudden mental fatigue and dyspnea, accompanied by sweating, dizziness and lower abdominal pain at 13 weeks of gestation. Her menstrual cycle was regular. She was diagnosed with SLE in 2013 and had been treated with glucocorticoid steroids (GC, Shangyao Xinyi Pharmaceutical Co., Ltd, Prednisone Acetate Tablets, Shanghai, China) and hydroxychloroquine (Shangyao Traditional and Western Pharmaceutical Co., Ltd, hydroxychloroquine sulfate tablets, Shanghai, China) since then. In December 2020, she experienced missed abortion combined with infection and massive vaginal hemorrhage. In order to control hemorrhage, she underwent interventional embolization of uterine artery and ultrasound guided curettage. At the same time, she accepted blood transfusion therapy. Because the transvaginal color Doppler ultrasound (TVCD) revealed residual uterine cavity and intrauterine adhesion, the patient accepted hysteroscopy 3 months later after curettage. During the hysteroscopy, massive bleeding of uterine cavity happened again. The patient underwent compression hemostasis using a FOLEY catheter and blood transfusion therapy. Because TVCD still revealed intrauterine adhesion, she underwent another hysteroscopy to incise adhesions and place an intrauterine device in the uterine cavity in March 2022. Two months later, she underwent hysteroscopy again to remove the device. After experiencing failure in conceiving for one and a half year, she decided to adopt in vitro fertilization embryo transfer in July 2022. In September 2022, she experienced controlled ovary hyperstimulation and got 7 available embryos. She accepted freezing-all due to uterine cavity effusion. In October 2022, TVCD revealed endometrial polyps and she underwent hysteroscopy again. In December 2022, she was transferred 2 blastocysts and confirmed intrauterine singleton pregnancy later. She had no discomfort until the 13 weeks of gestation (Table 1).

**Table 1:** The treatment process of the patient.

2013	Diagnosed as SLE
December 2020	Missed abortion, curettage
March 2021	Hysteroscopy + FOLEY catheter to compress
March 2022	Hysteroscopy, place device
May 2022	Hysteroscopy, remove device
October 2022	Hysteroscopy, revealed endometrial polyps
September 2022	Retrieval ovum
October 2022	Embryo transfer
February 2023	13 weeks of gestation, mental fatigue, dyspnea

SLE = systemic lupus erythematosus.

The vital signs were as follows: body temperature, 35.8 °C; blood pressure, 86/61 mm Hg; pulse, 127 beats per min; respiratory rate, 21 breaths per min. The patient showed anemia, poor mental state, clear consciousness and had response to stimulus. She had slight abdominal tenderness and cervical lifting pain.

Blood analysis revealed anemia (hemoglobin [Hb], 9.7 g/dL; red blood cell [RBC], 3.42*10^12^/L). Coagulation function was normal, with DD dimer 9190 μg/L. We performed antishock treatment such as fluid replacement and volume expansion. The blood analysis was checked after 3 hours which showed deterioration of anemia (Hb 6.6 g/dL, RBC 2.33*10^12^/L). Coagulation function were checked which showed coagulation disorders (prothrombin time, 24.0 seconds; activated partial thromboplastin time, >100 seconds; thrombin time, >80 seconds; fibrinogen,132 mg/dL).

**Figure 1.**
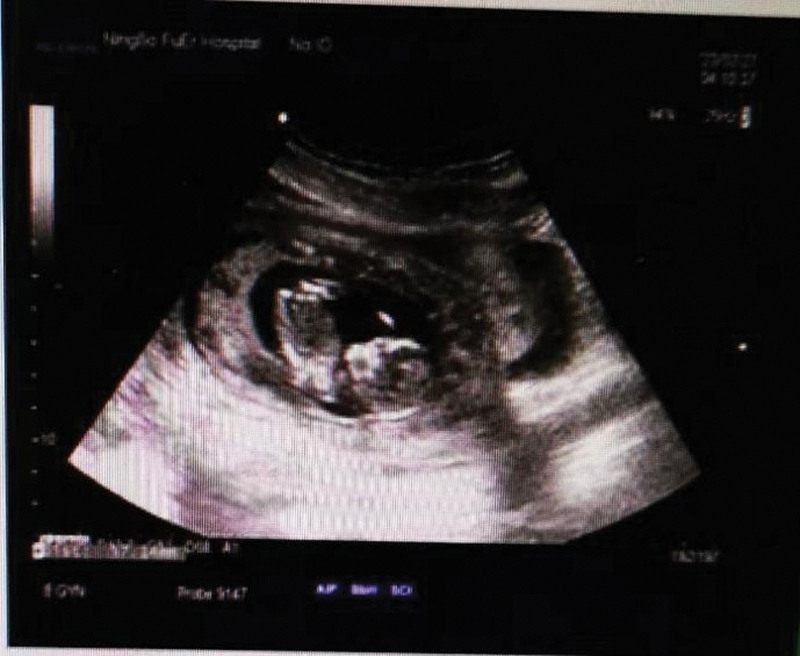
Ultrasound, fetus in the uterus. Liquid was stored in anterior recess and right iliac fossa.

**Figure 2.**
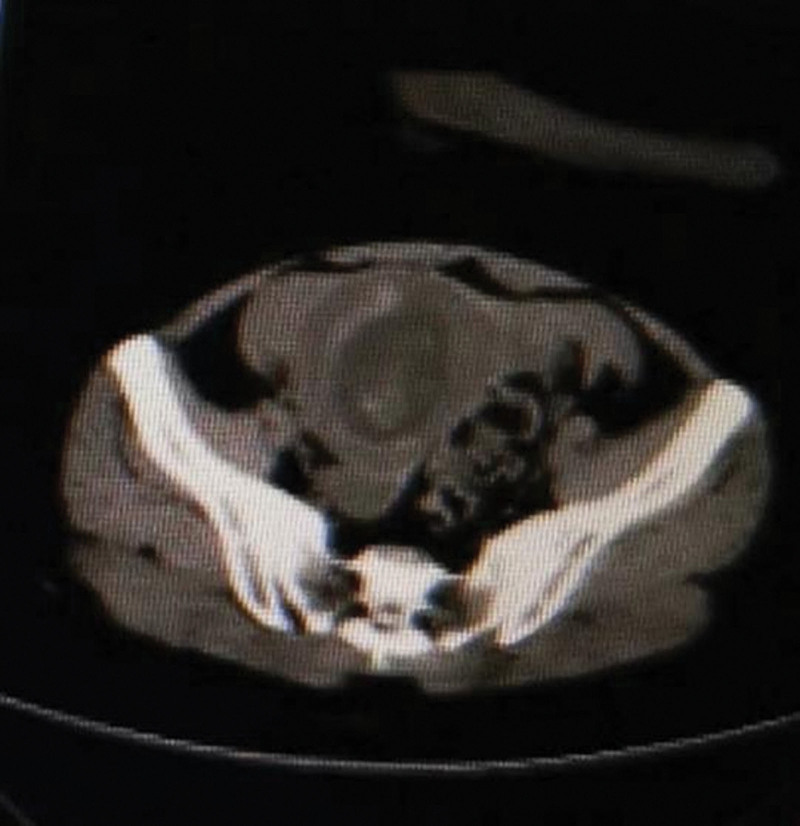
Plain CT scan, fetus in the uterus. A mount of liquid was stored in abdominal cavity.

Ultrasonography revealed one fetus with heartbeat in gestational sac properly located. The size of sac was 69 × 44 × 52 mm and fetal crown-rump length was 57 mm. There was effusion in anterior recess and right iliac fossa, 34 mm and 27 mm respectively (Fig. 1). Plain computed tomography scan of the upper abdomen showed a large amount of fluid accumulating in the abdominal cavity, intrauterine early pregnancy and intrahepatic calcification (Fig. 2).

**Figure 3.**
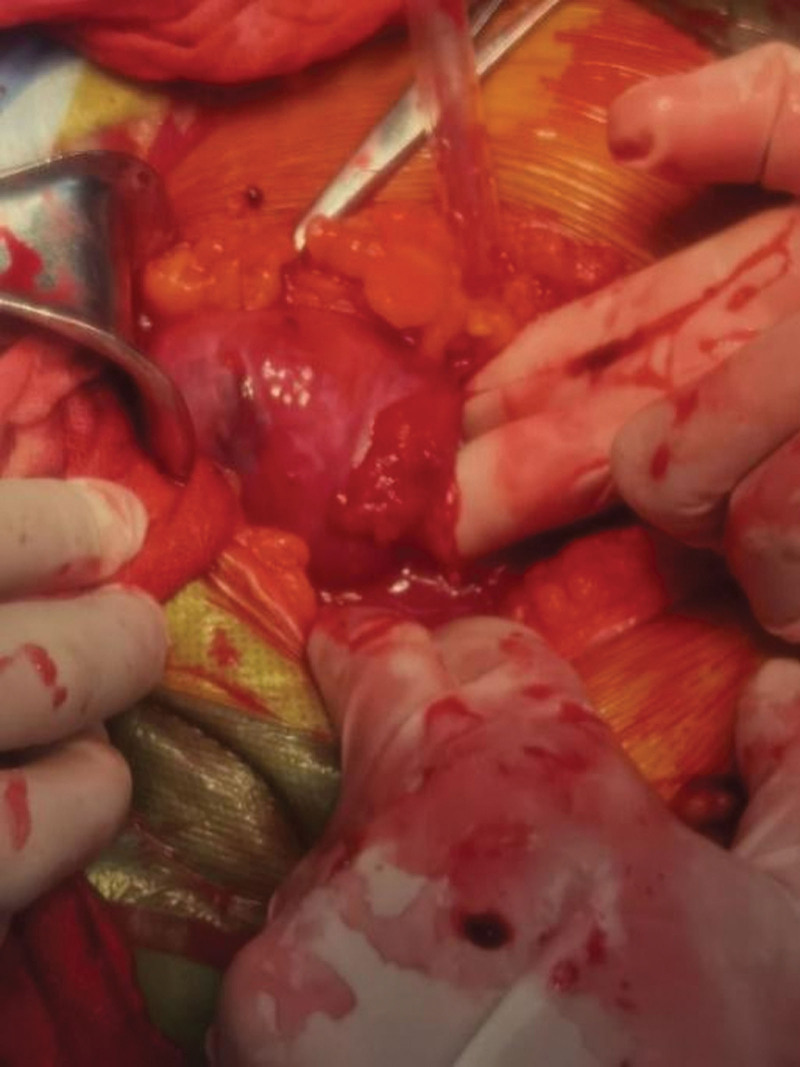
The breach is in the posterior wall of uterus. The purplish site was weak myometrium.

An emergent laparotomy was performed to find the origin of bleeding under general anesthesia while transfusion of leukocyte removed suspension red blood cells and fresh frozen plasma. There was massive blood accumulation and clot in the abdominal cavity. Multiple myometrium was weak and appeared purplish blue, with only the serous layer visible. There was an irregular breach about 2.5 cm long in the posterior wall, with exposed placenta and villous tissue (Fig. 3).

Based on the patient’s history, symptoms, signs, laboratory, and laparotomy finding, she was diagnosed with: UR, hemorrhagic shock, SLE, and intrauterine adhesion. She received repairing the myometrial rupture and removing fetus. She underwent autotransfusion of about 820 mL and transfusion of 1200 mL of leukocyte removed suspension red blood cells, 950 mL fresh frozen plasma, 20 U cryoprecipitate, 10 U platelet and 45 g albumin in total. Blood analysis revealed improving of anemia (Hb 7.2 g/dL) and coagulation function after operation. The Hb rechecked showing decline (Hb 6.5 g/dL) in 2 hours after surgery and becoming lower in 6 hours (4.3 g/dL). Ultrasonography rechecked which revealed pelvic and peritoneal effusion. The patient underwent transfusion of red blood cells and fresh frozen plasma again. After about 12 hours after surgery, the patient had abdominal distension and pain with dyspnea. Her blood pressure, pulse and breathe were 114/70 mm Hg, 170 beats/min, and 30 breaths/min, respectively. The Hb was 5.1 g/L at the moment. Considering persistent intraperitoneal hemorrhage, the patient underwent laparotomy again. A large amount of dark red blood accumulation and clot was observed in the pelvic and abdominal cavity. The uterus was soft with multiple active bleeding at the suture sites. After obtained the consent of the patient’s husband, we performed the operation of subtotal hysterectomy. The histopathologic examination showed multiple blood clots in the myometrium with a rupture at the fundus. Postoperative recovery was uneventful. She was discharged on the 8th day after operation.

## 3. Discussion

UR is a disastrous event during pregnancy. It was reported that the incidence of UR is 5.1 per 10,000 deliveries in a scarred uterus, and approximately 0.45 to 0.7 per 10,000 deliveries in an unscarred uterus.^[12,13]^ Most cases of UR occur in late pregnancy or during delivery, rarely in first or early second trimester. The most common symptoms of UR include sudden onset of severe abdominal pain, fetal distress, vaginal bleeding and maternal hypovolemic shock.^[14]^ The patient had the typical symptoms and signs of hypovolemic shock accompanied with lower abdominal pain at 13 weeks of gestation, which was not difficult to obtain the diagnosis of UR.

The primiparous patient had no history of uterine scarring or other known risk factors for UR. Why it happened? She had the underlying disease of SLE and took GC for several years, both of which may be related to UUR. Joseph et al reported spontaneous rupture of an unscarred uterus at 23 weeks which related to the past history of SLE and long-term oral prednisolone.^[15]^ In that case, the woman suffered from SLE for 19 years and conceived a twin gestation after IVF. After a subtotal hysterectomy, they found focal area of placental chorionic plate infiltrating into partial thickness of the myometrium by histopathologic examination. Namita et al also presented a case of marked thinning of the myometrium in a woman who had a history of SLE for 9 years.^[16]^ The woman was found to have placenta accreta and placenta previa and she chose an elective lower uterine segment cesarean at 36 wk + 5 d gestation. She experienced peripartum hysterectomy because of significant bleeding. Histologic examination showed marked thinning of the myometrium which was <1 mm thick in most of the uterus except for lower uterine segment. All of the 3 patients had the history of SLE and took long-term GC, but the resultant difference of uterine structural change existed. In the present case, the past history of SLE was 10 years and the occurrence of UR at 13 weeks of gestation. The marked thinning of the myometrium was similar with Namita’s report except the absence of placenta accreta and placenta previa.

How does the disease of SLE affect the myometrium? A clinical trial shows that estradiol concentrations were found to be significantly lower in pregnant women with SLE throughout pregnancy when compared with healthy pregnant women.^[17]^ Antiestrogen receptor α (anti-ERα) autoantibodies that interfere with T lymphocyte homeostasis were present in 45% of the patients with SLE.^[18]^ The lower estrogen levels and the presence of anti-ERα autoantibodies may have contributed to insufficient growth of the uterus that it can’t accommodate the rapid fetal growth, resulting in UR. However, there were no studies so far that directly measure the effects of SLE on the integrity of uterine smooth muscle. Long-term glucocorticoid treatment may decrease the concentration of estradiol receptor and weaken the estradiol-stimulated growth of uterus.^[19]^ Furthermore, long-term glucocorticoid use weakens muscle and induces atrophy of smooth muscle by decreasing protein synthesis.^[20,21]^ Emmy et al presented a UUR of multigravida pregnant woman with chronic use of glucocorticoids for the disease of psoriatic arthritis, which further proved a causal link between the chronic use of GCs and UR.^[2]^

In the present case, besides the past history of SLE and long-term use of GC, curettage and 4 times hysteroscopy may also contribute to the occurrence of UUR. Because these intrauterine surgeries may cause an unrecognized uterine injury or even perforation. There was a UR case report during pregnancy caused by previous curettage.^[6]^

Repairing the myometrial rupture is effective to stop bleeding in cases of UR. There was a case report of continuing pregnancy until successfully delivered by cesarean section at 34 weeks of gestation after repairing the myometrial rupture.^[22]^ In the present case, after the first time of laparoscopy to repair the myometrial rupture and remove gestational tissue, there was multiple active bleeding at the suture sites. Subtotal hysterectomy performed in the second laparoscopy in order to save the patient’s life. The reason of failure in preserving the uterus is the marked thinning of the myometrium and destruction of contractility.

In conclusion, we present a very rare case of UUR at 13 weeks of gestation. The underlying disease of SLE, chronic use of corticosteroids and intrauterine operations may lead to UR. Identifying these risk factors can help with quick diagnosis and management of UR.

## Acknowledgments

We would like to thank our colleagues at the Department of Reproduction Medicine Center of Ningbo Women and Children’s Hospital.

## Author contributions

**Funding acquisition:** Liming Zhou, Juan Zheng.

**Supervision:** Juwei Hu.

**Validation:** Liming Zhou.

**Writing – original draft:** Juan Zheng.

**Writing – review & editing:** Juan Zheng, Juwei Hu.
